# Altered Gene Expression of IL-35 and IL-36α in the Skin of Patients with Atopic Dermatitis

**DOI:** 10.3390/ijms25010404

**Published:** 2023-12-28

**Authors:** Weronika Zysk, Krzysztof Sitko, Stefan Tukaj, Anna Zaryczańska, Magdalena Trzeciak

**Affiliations:** 1 Department of Dermatology, Venereology and Allergology, Medical University of Gdansk, 80-214 Gdansk, Poland; weronikazysk@gumed.edu.pl (W.Z.); anna.zaryczanska@gumed.edu.pl (A.Z.); 2 Department of Molecular Biology, Faculty of Biology, University of Gdansk, 80-308 Gdansk, Poland; krzysztof.sitko@phdstud.ug.edu.pl (K.S.); stefan.tukaj@ug.edu.pl (S.T.)

**Keywords:** atopic dermatitis, biomarker, tape stripping, skin immunology

## Abstract

A comprehensive understanding of atopic dermatitis (AD) pathogenesis is desired, especially in the current era of novel biologics and small molecule drugs. In recent years, new cytokines have emerged that may play a significant role in the pathogenesis of AD. Using the tape stripping (TS) method, this study analyzed the gene expression of IL-35 and IL-36α in lesional and nonlesional AD skin compared with healthy skin and their association with the clinical features of AD among the Polish population. Ten AD patients and seven healthy individuals were enrolled. The lesional skin of the AD patients showed significantly higher expression levels of IL-35 compared to healthy skin (*p* = 0.0001). The expression level of IL-36α was significantly higher in lesional AD skin than in nonlesional AD skin (*p* = 0.0039) and healthy skin (*p* = 0.0045). There was a significant negative correlation between AD severity and the expression level of IL-35 in both lesional (R = −0.66, *p* = 0.048) and nonlesional skin (R = −0.9, *p* = 0.0016). In summary, both IL-35 and IL-36α appear to play a role in the pathogenesis of AD. Furthermore, it might be speculated that IL-35 and IL-36α may be potential candidates for disease biomarkers. However, further studies are needed to verify these assumptions and comprehensively elucidate their importance in the pathogenesis of AD.

## 1. Introduction

Atopic dermatitis (AD) is a chronic relapsing inflammatory skin disease, characterized by a highly complex pathogenesis determined by altered immune response, environmental and genetic factors, and insufficiency of the skin barrier function [1]. AD is associated with a higher prevalence not only of allergic comorbidities but also non-allergic conditions, such as obesity, cardiovascular disease, and autoimmune disease [2] The disease significantly reduces the quality of life and emotional well-being of the patients. The burden of the disease includes, among others, burdensome itch, skin pain, sleep disturbance, psychosocial distress, stigma, functional disturbances, and limited activities of daily living. Patients with AD are more likely to suffer from depression, anxiety, and suicidality, which is highly correlated with disease severity [3]. Moreover, AD is a very heterogeneous disease that encompasses a variety of endotypes and phenotypes orchestrated by the patient’s age at onset, race and ethnicity, disease chronicity, and IgE levels [4]. From a molecular point of view, specific immune pathway contributions and different characteristics of skin barrier alterations drive specific endotypes and phenotypes of AD [4]. Complicated and not fully understood immunological phenomena in AD prompt detailed and intensive research. The better we understand the pathogenesis of AD, the better we can treat it. A comprehensive understanding of AD pathogenesis is exciting and desired, especially in the current era of novel biological drugs and small molecules that address immunological disorders while simultaneously improving the epidermal barrier function and restoring skin microbiome homeostasis through increasing microbial diversity and decreasing *Staphylococcus aureus* colonization [5]. Dupilumab, human anti-interleukin-4 receptor α monoclonal antibody, was the first biologic drug approved for patients with moderate-to-severe AD. The approval of it marked a significant milestone in the treatment of these patients. Dupilumab has been shown to be a safe and effective treatment for moderate-to-severe AD, offering long-term efficacy, improvement of sleep outcomes, and rapid relief from pruritus [6,7]. Moreover, novel treatment of AD can significantly improve the quality of life and psychological condition of patients. Continuous treatment with dupilumab for up to three years has been shown to provide long-term improvements in the psychological well-being of patients with moderate-to-severe AD [8]. Furthermore, it has been shown that the treatment of AD with this drug has similar efficacy and safety in patients with and without comorbidities [9]. Intensive research into the pathogenesis of AD, which has been conducted over the past few years, constitutes the foundations for the search for new therapeutic targets. In recent years, several novel cytokines have been suggested, which can potentially play a significant role in the pathogenesis of AD [10].

IL-35 is an anti-inflammatory cytokine belonging to the IL-12 family. It is recognized as a cytokine pivotal in maintaining immune homeostasis. It exerts immunomodulatory properties by inducing a unique population of regulatory T cells releasing IL-35, termed iTr35, as well as IL-10-producing regulatory B cells (IL-10+Bregs) and IL-35-producing regulatory B cells (IL-35+Bregs), while suppressing Th1, Th17, and Th2 cell responses [11]. Due to its interesting immunomodulatory properties, there is a growing interest in its significance in many immune-related diseases [12]. So far, two studies have been conducted to assess only serum levels of IL-35 in AD patients. The results of these studies are conflicting. The first study involving AD infants showed increased serum levels of IL-35 in them [13], while the other study showed decreased serum levels of IL-35 in AD individuals compared to healthy [14]. Thus, the role of IL-35 in the pathogenesis of AD remains enigmatic.

Unlike IL-35 with anti-inflammatory properties, the IL-36 subfamily, including pro-inflammatory agonists IL-36α, IL-36β, and IL-36γ, and one antagonist, IL-36Ra, is recognized as a key initiator of inflammation in the skin [15]. Human keratinocytes are the main source of IL-36 cytokines in the skin, particularly after stimulation with TNFα, IL-17, IL-22, and IL-1β [15]. Then, IL-36 cytokines can promote pro-inflammatory cytokines, creating a positive feedback loop [15]. The first reports underlined the role of the IL-36 pathway in psoriasis—the representative Th17-dominant disease [15]. However, growing evidence suggests that the IL-36 pathway may also be involved in AD pathogenesis [16,17,18,19,20,21]. Although AD is predominantly Th2-driven, involvement of Th1 and Th17 cells is also observed depending on endotypes/phenotypes of AD [1]. IL-36 cytokines have been described to regulate IFN-γ, IL-17, and IL-4 production and to induce chemokine expression in keratinocytes. Thus, they may influence the immune milieu of inflamed AD skin [22].

Due to the not fully elucidated role of IL-35 and IL-36α cytokines in the pathogenesis of AD, we decided to take a closer look at them with the use of a minimally invasive method called tape stripping (TS). This method allows the collection of samples of stratum corneum (SC) and some stratum granulosum (SG) using adhesive tapes [23]. It can effectively identify multiple biological entities, including proteins, proteases, lipids, and RNA, enabling the assessment of a diverse array of immune and epidermal barrier biomarkers in both lesional and nonlesional skin [24]. The TS technique is simple, painless, and does not cause bleeding or scarring, making it a promising and reliable alternative to skin biopsies [25].

The majority of studies concerning interleukins in the context of understanding AD pathogenesis are based on their concentration in the blood serum. Recently, the issue has been raised that the phenomena occurring locally in the skin play a crucial role in the pathogenesis of AD as a disease mainly of the skin. An ideal example is the recent reports of IL-4 as a centrally acting cytokine, while IL-13 exerts its peripheral effects at the tissue level, significantly influencing skin biology in patients with AD [26].

Therefore, using the TS method, we aimed to investigate the gene expression of IL-35 and IL-36α in lesional and nonlesional AD skin compared with the skin of healthy controls and their association with the clinical features of AD among the Polish population.

## 2. Results

### 2.1. Demographic Data

A total of 10 patients, 4 females (40%) and 6 males (60%), with a mean age of 26 ± 13.5 years (range 11–54 years) were enrolled. According to the SCORAD scale, a mild course of AD was observed in 3 patients (33.3%), a moderate course in 5 patients (55.6%), and a severe course in 1 patient (11.1%). Regarding the EASI score, 4 patients (40%) had moderate, 5 (50%) had severe, and 1 (10%) had very severe AD. The mean pruritus severity and sleep loss were 6.0 ± 2.05 and 5.4 ± 2.8, respectively. Atopic comorbidities include allergic rhinitis in 5 patients (50%), allergic conjunctivitis in 1 patient (10%), and asthma in 5 patients (50%). A total of 3 patients (30%) had no atopic comorbidity. The eosinophilia was noted in 5 patients out of 9 (55.6%). The control group consisted of 7 subjects: 3 females (42.9%) and 4 males (57.1%), with a mean age of 30 ± 5.6 years (range 27–43 years) (Table 1).

### 2.2. The Expression Level of IL-35 and IL-36α in Tape Strips from Lesional AD Skin, Nonlesional AD Skin, and Healthy Control Skin

**Figure 1:**
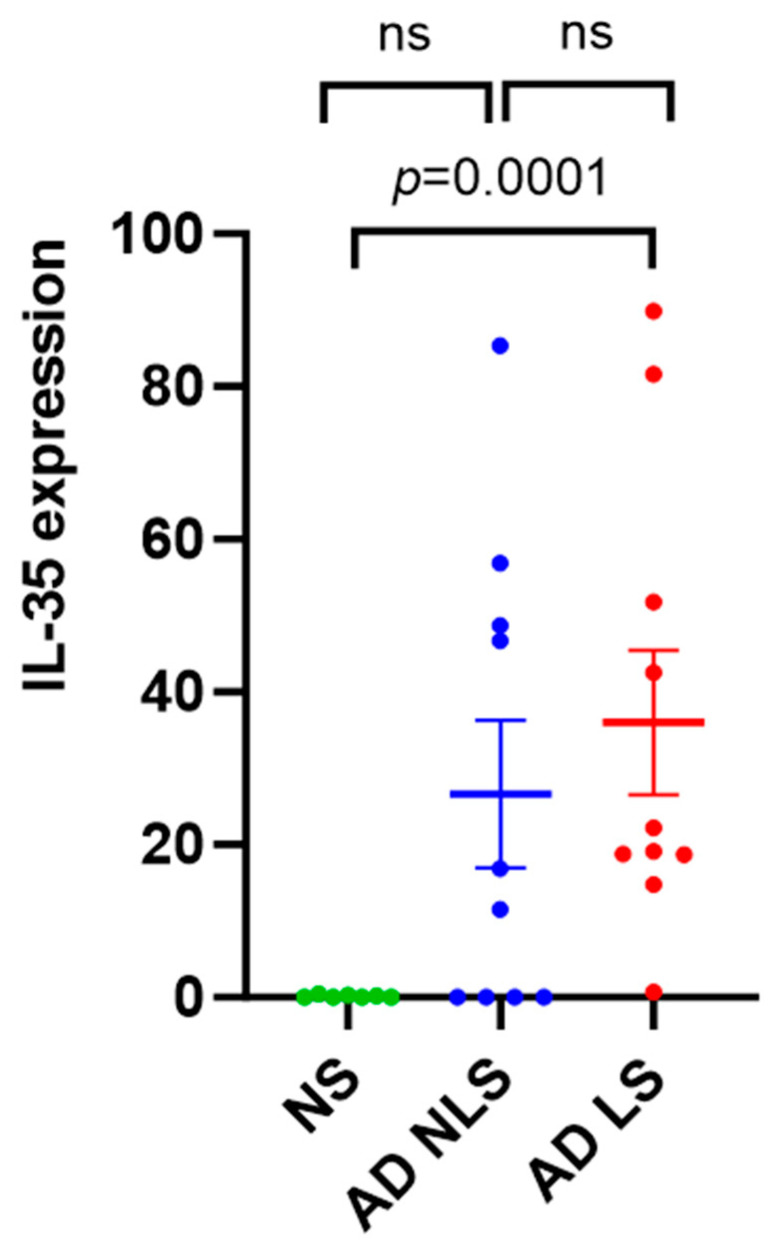
qRT-PCR analysis of IL-35 gene expression in AD skin vs. normal skin. Data are presented as mean ± SEM. Black lines indicate the significance of the comparison between AD skin and normal skin. ns: non-significant; AD: atopic dermatitis; NS: normal skin; NLS: nonlesional skin; LS: lesional skin.

The lesional skin of AD patients showed significantly higher expression levels of IL-35 compared to healthy skin (*p* = 0.0001). There was no significant difference in the expression levels of IL-35 between the nonlesional AD skin and healthy skin (*p* = 0.2012) or between the lesional and nonlesional AD skin (*p* = 0.3223) (Figure 1).

**Figure 2:**
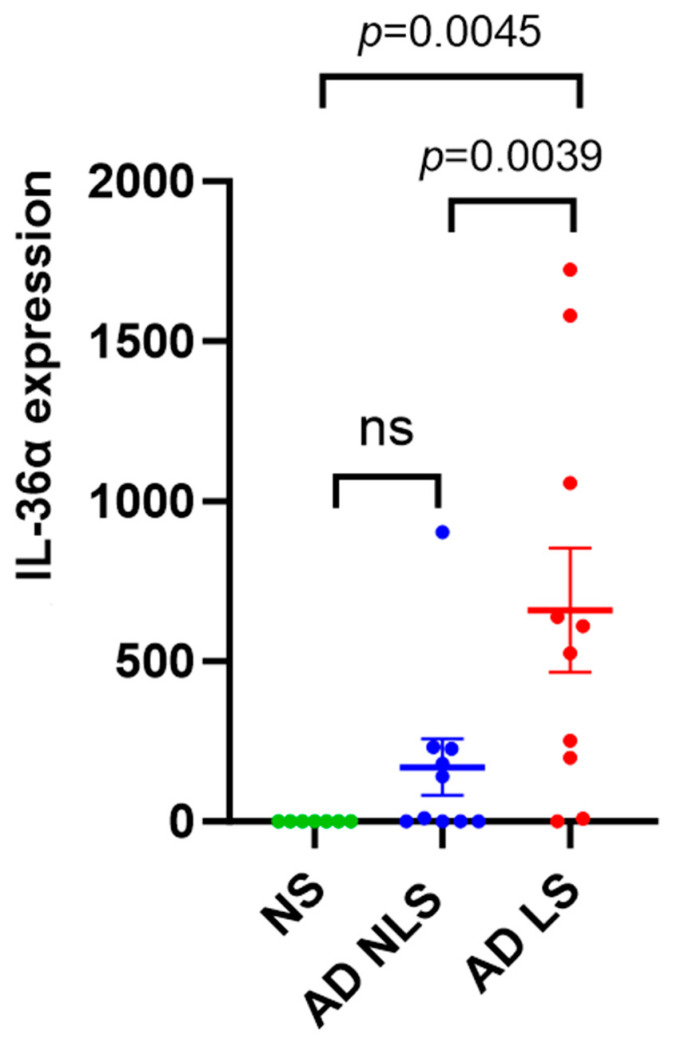
qRT-PCR analysis of IL-36α gene expression in AD skin vs. normal skin. Data are presented as mean ± SEM. Black lines indicate the significance of the comparison between AD skin and normal skin. ns: non-significant; AD: atopic dermatitis; NS: normal skin; NLS: nonlesional skin; LS: lesional skin.

The expression level of IL-36α was significantly higher in lesional AD skin than in nonlesional AD skin (*p* = 0.0039) and healthy skin (*p* = 0.0045). No significant difference in the expression levels of IL-36α between the nonlesional AD skin and healthy skin was found (*p* = 0.3505) (Figure 2). Healthy skin was characterized by weak expression of both IL-35 and IL-36α.

### 2.3. Correlations of the Expression Level of IL-35 and IL-36α with Clinical Features of AD

The expression level of IL-35 showed a significant negative correlation with AD severity measured by EASI in both lesional (R = −0.66, *p* = 0.048) and nonlesional skin (R = −0.9, *p* = 0.0016). Regarding SCORAD, a significant negative correlation with the expression levels of IL-35 in nonlesional skin (R = −0.89, *p* = 0.004) and a trend toward significant in lesional skin (R = −0.58, *p* = 0.1) were observed. In both lesional and nonlesional AD skin, there was no relationship between IL-35 expression levels and eosinophilia, allergic rhinitis, allergic conjunctivitis, asthma, pruritus, and sleep problems.

Regarding IL-36α, we observed no correlation between its expression and SCORAD, EASI, eosinophilia, allergic rhinitis, allergic conjunctivitis, asthma, pruritus, or sleep problems in either lesional or nonlesional AD skin (Table 2).

## 3. Discussion

At first sight, increased IL-35 expression as an anti-inflammatory cytokine in lesional AD skin, observed in our study, may seem unexpected. However, diving into the details, the pro-inflammatory milieu has been described to upregulate the mRNA expression of p35 and EBI3 subunits, which form IL-35. Pro-inflammatory cytokines such as tumor necrosis factor-α (TNF-α), interferon-γ (IFN-γ), and IL-1β induce upregulation of IL-35 [27]. Depending on the chronicity of AD skin lesions, different intensity of infiltration of the above-mentioned pro-inflammatory cytokines is observed [28]. Enhanced expression of IL-1β is characteristic of the acute phase of AD, occurring within the first 72 h after lesion onset. The expression of Th1–related cytokines (IFN-γ, TNF-a) intensifies during the chronic phase of AD [4,29]. However, acute and chronic lesions of AD often overlap in the same individual [28]. This explains why, in our study, the expression level of IL-35 was increased in lesional AD skin, characterized by high-level inflammation, compared to healthy control skin. Comparing nonlesional AD skin with both healthy control skin and lesional AD skin, we found no significant differences in IL-35 expression. However, in general, we observed an upward trend of its expression toward lesional AD skin. This may result from the fact that the nonlesional skin of AD patients shows signs of low-level inflammation, with lower infiltration of pro-inflammatory cytokines than is observed in lesional skin [28]. It may be hypothesized that under strong inflammatory conditions, IL-35 increased expression to try to compensate for the pro-inflammatory responses. To the best of our knowledge, this is the first study evaluating the IL-35 expression in the skin of AD patients. In another immune-related disease, psoriasis, Owczarczyk-Saczonek et al. found, in contrast to our results, reduced expression of IL-35 in psoriatic lesions compared to perilesional lesions and healthy skin. The authors hypothesized that it may be due to IL-35 wearing off during the course of inhibiting inflammation [30]. On the other hand, differences in IL-35 expression between these two conditions suggest the potential use of IL-35 as a biomarker to differentiate challenging cases of psoriasis and AD. Furthermore, we found a negative correlation between IL-35 expression in lesional and nonlesional skin and AD severity. Similar to our results, Kiwan et al. observed a negative correlation between IL-35 serum level and the severity of the disease assessed by the SCORAD scale [14]. Moreover, the expression of IL-35 may be a potentially useful clinical biomarker reflecting the severity of AD. Additionally, considering that nTregs are the main source of IL-35 [11], it seems that it may have the greatest effect at sites of strong nTregs activation. Although there is conflicting data about Tregs frequencies in AD [31], increased numbers of CD4+CD25+FOXP3+ Tregs (nTregs) were shown in lesional skin biopsies of patients with AD [32]. Roesner et al. suggested that Tregs in AD are activated, but the inflammatory milieu can hinder their function [33]. Whether Tregs are reduced in AD or are present but fail to function optimally remains to be elucidated.

In line with a previous study by Suarez-Farinas et al. [18], we found increased expression of IL-36α in lesional AD skin compared to healthy skin as well as nonlesional AD skin. In nonlesional AD skin versus healthy skin, increased expression of IL-36 isoforms including IL-36α was not generally observed [17,18,19], which is also consistent with our results. However, contrastingly, in Tanzanian AD patients, IL-36α was upregulated in both lesional and nonlesional skin [20]. Expression of IL-36α has been found to be induced upon the exposure of IL-17A, TNF-α, and IL-22 [34]. Although AD lesions are primarily Th2-driven, Th22 skewed with the overproduction of IL-22 is also observed, while Th1 and Th17 upregulation varies depending on AD endotype/phenotype [4]. S. aureus, commonly found on the skin of AD patients, triggers IL-36α expression in the epidermis [21]. The prevalence of S. aureus in AD patients is higher on lesional skin, with reported rates of 70% compared to 39% on nonlesional skin [35]. This fact, among others, may contribute to our observed high expression of IL-36α in skin lesions. Furthermore, it was demonstrated that IL-36 leads to decreased expression of filaggrin, which may exacerbate barrier deficiencies and is well known to be one of the major features of AD [15]. We did not note any significant correlation between IL-36α expression and SCORAD or EASI. Perhaps this results from a small group of patients in our study. Recently, a modest efficacy of an anti-IL-36 receptor antibody in patients with moderate-to-severe AD was demonstrated in a clinical trial, suggesting that the IL-36 pathway does not play a major role in the pathogenesis of AD [36]. However, given that IL-36α has been found to regulate mostly Th17 immunity [37], it may be hypothesized that it may be more significant in endotypes/phenotypes of AD with enhanced Th1 and Th17 inflammation. Comparing the expression in extrinsic and intrinsic AD, IL36α, IL36γ, and IL36Ra were more increased in intrinsic AD characterized by enhanced Th1 and Th17 activation [15]. Of note, the significant difference in IL-36α expression between lesional and nonlesional skin observed in our study suggests the possibility of IL-36α as a potential biomarker to discriminate lesional from nonlesional skin and may be helpful in the monitoring of the improvement of skin lesions in AD patients.

To the best of our knowledge, this is the first study of its kind conducted on Polish patients. The findings of this study can serve as a valuable point of reference not only within Poland but also in Central Europe, as no similar research has been undertaken in this area thus far.

The major limitation of this study is the limited number of study participants. However, most of these studies are led in small groups [38,39,40,41]. No correlation between the expression level of IL-36α and the investigated clinical features of AD may result from the small number of patients in our study. Another important fact is that our results are representative of only Caucasian people. However, in the context of ethnic differences in AD, this may be additional value as well. Given the high heterogeneity of AD, it is important to consider the potential variability of results across different populations. Additionally, TS itself has some limitations. The potential differences in the depth of epidermal TS among samples may result in differential expression of some genes [42]. Therefore, it is important to consider this fact during the interpretation of the obtained results amongst different labs. Our method was performed according to the same protocol.

To conclude, the lesional skin of AD patients was characterized by increased expressions of IL-35 and IL-36α compared to healthy skin, which indicates a significant role of both cytokines in the pathogenesis of AD. According to our results, it appears that IL-35 plays an anti-inflammatory role in AD, and its increased expressions in lesional skin were most probably due to an attempt to compensate for inflammation. The significance of the IL-36α pathway in AD pathogenesis may depend on the AD endotype/phenotype due to its major involvement in Th1/Th17-related inflammation [15]. Therefore, further investigations involving exactly phenotyped AD patients are necessary to comprehensively elucidate the importance of IL-36α in the pathogenesis of AD. Furthermore, it might be speculated that IL-35 and IL-36α may be candidates for biomarkers, which are extremely desirable, especially in the current era of more targeted therapies for AD. IL-35 may be a potential biomarker inversely correlated with the severity of AD in both lesional and nonlesional skin. Evaluating the exact and objective severity of the disease seems to be crucial for the possibility of planning the appropriate time, as well as the intensity, of AD treatment. When it comes to IL-36α, it may be a potential biomarker to discriminate lesional from nonlesional skin, thereby evaluating therapeutic responses. Importantly, the TS method provides a minimally invasive approach to tracking therapeutic response. However, the proposition of IL-35 and IL-36α as potential candidates for biomarkers must be approached with caution at this point, as our findings are based only on transcriptomic analysis. Confirming gene expression at the protein level would enhance the reliability and utility of biomarker proposal. Thus, it is recommended that future investigations integrate both RNA and protein analyses. Additionally, this approach will provide a more comprehensive understanding of the molecular changes in AD. Undoubtedly, further studies appropriately designed, involving larger cohorts of patients, are needed to verify our observations.

## 4. Material and Methods

### 4.1. Patients

The study included 10 AD patients recruited from the outpatient clinics of Dermatology, Venereology, and Allergology at the Medical University of Gdańsk, based on AD diagnosis criteria proposed by Hanifin and Rajka [43], and 7 healthy individuals with no medical history of allergy, autoimmune diseases, or malignancies. Both AD patients and healthy individuals were Caucasian. Both groups were age–sex–ethnicity-matched. Patients receiving immunosuppressive treatment, other immunotherapies, or UV therapy, and patients with clinical skin infections, were excluded from the study.

### 4.2. Determination of AD Severity

AD severity was assessed by the SCORAD (Severity Scoring of Atopic Dermatitis) and EASI (Eczema Area and Severity Index). In the SCORAD scale, AD severity is defined as mild (SCORAD < 25), moderate (SCORAD 25–50), and severe (SCORAD > 50) [44]. The proposed severity strata of AD for the EASI are as follows: clear (EASI 0), almost clear (EASI 0.1–1.0), mild (EASI 1.1–7.0), moderate (EASI 7.1–21.0), severe (EASI 21.1–50.0), and very severe (EASI 50.1–72.0) [45]. Pruritus severity and sleep problems were estimated using a visual analog/numeric rating scale of 0–10.

### 4.3. Tissue Sampling and Analysis

Among AD patients, 10 consecutive tape strips were collected from the lesional skin and nonlesional skin from nearby skin in the same anatomical region. The skin of healthy individuals was tape-stripped from the same areas. Tape strips were then kept frozen at −80 °C.

The quantitative real-time polymerase chain reaction (qRT-PCR) was applied to analyze the relative gene expression of IL-35 and IL-36α. Total RNA was isolated from frozen tape strips using RNeasy Mini Kit (QIAGEN, Hilden, Germany) and subsequently reverse-transcribed using QuantiTect Reverse Transcription Kit (QIAGEN), according to the manufacturer’s instruction. Obtained cDNA was analyzed using QuantiNova SYBR Green PCR Kit (QIAGEN) in LightCycler^®^ 480 Instrument II (Roche, Basel, Switzerland). Two reference genes, GAPDH and BACT, were selected using BestKeeper^©^ software (version 1.0). Primers used in this study:

IL-35: forward, 5′-CTGGATCCGTTACAAGCGTCAG-3′ and reverse, 5′-CACTTGGACGTAGTACCTGGCT-3′

IL-36α: forward, 5′-CTTCAGGACCAGACGCTCATAG-3′ and reverse, 5′-GGCAGAGATTGAGTCCATTCAGG-3′

GAPDH: forward, 5′-GTCTCCTCTGACTTCAACAGCG and reverse, 5′-ACCACCCTGTTGCTGTAGCCAA-3′

BACT: forward, 5′-CACCATTGGCAATGAGCGGTTC-3′ and reverse, 5′-AGGTCTTTGCGGATGTCCACGT-3′

### 4.4. Statistical Analysis

Statistical analyses were performed using GraphPad Prism 10 software (GraphPad, San Diego, CA, USA). The difference in the relative gene expression level of IL-35 and IL-36α between lesional and nonlesional AD skin was determined by the Wilcoxon signed-rank test. The Mann–Whitney U test was used to compare the relative gene expression level of IL-35 and IL-36α between healthy skin and both lesional skin and nonlesional skin. Correlations between gene expression and clinical features of AD were evaluated by Spearman correlation coefficients. *p* < 0.05 was considered statistically significant.

## Figures and Tables

**Table 1:** Demographic and clinical characteristics of study participants.

Characteristic	AD Patients (*n* = 10)	Healthy Controls (*n* = 7)
Sex, *n* (%)		
Female	4 (40.0%)	3 (42.9%)
Male	6 (60.0%)	4 (57.1%)
Age, mean ± SD	26 ± 13.5	30 ± 5.6
AD severity according to SCORAD, *n* (%)		-
Mild (SCORAD < 25)	3 (33.3%)	
Moderate (SCORAD 25–50)	5 (55.6%)	
Severe (SCORAD > 50)	1 (11.1%) ^1^	
AD severity according to EASI, *n* (%)		-
Clear (EASI 0)	0 (0.0%)	
Almost clear (EASI 0.1–1.0)	0 (0.0%)	
Mild (EASI 1.1–7.0)	0 (0.0%)	
Moderate (EASI 7.1–21.0)	4 (40.0%)	
Severe (EASI 21.1–50.0)	5 (50.0%)	
Very severe (EASI 50.1–72.0)	1 (10.0%)	
Pruritus, mean ± SD	6.0 ± 2.05	-
Sleep loss, mean ± SD	5.4 ± 2.8	-
Atopic comorbidities, *n* (%)		-
Allergic rhinitis	5 (50.0%)	
Allergic conjunctivitis	1 (10.0%)	
Asthma	5 (10.0%)	
Eosinophilia, *n* (%) *		-
Yes	5 (55.6%)	
No	4 (44.4%) ^2^	

^1^ One patient was not assessed in SCORAD scale; percentage data were calculated assuming *n* = 9 in this case. ^2^ The eosinophil count was not assessed in one patient; percentage data were calculated assuming *n* = 9 in this case. * Eosinophilia was defined as a total peripheral blood eosinophil count > 0.45 × 10^9^/L.

**Table 2:** Correlations between IL-35 and IL-36α genes expression and clinical features of AD.

	SCORAD	EASI	VAS	Sleep	Eosinophilia	AR	AC	Asthma
Nonlesional IL-35	R = −0.89	R = −0.90	R = −0.33	R = −0.22	R = 0.52	R = 0.03	R = −0.06	R = −0.03
	*p* = 0.004	*p* = 0.002	*p* = 0.358	*p* = 0.540	*p* = 0.190	*p* = 1.0	*p* = 1.0	*p* = 1.0
Lesional IL-35	R = −0.58	R = −0.66	R = −0.33	R = −0.60	R = 0.17	R = −0.10	R = −0.29	R = 0.17
	*p* = 0.115	*p* = 0.048	*p* = 0.348	*p* = 0.072	*p* = 0.730	*p* = 0.841	*p* = 0.60	*p* = 0.690
Nonlesional IL-36α	R = 0.02	R = −0.04	R = −0.18	R = −0.006	R = 0.0	R = 0.10	R = −0.41	R = −0.59
	*p* = 0.980	*p* = 0.932	*p* = 0.623	*p* = 0.993	*p* = 1.0	*p* = 0.841	*p* = 0.40	*p* = 0.095
Lesional IL-36α	R = 0.13	R = 0.02	R = −0.28	R = 0.32	R = 0.35	R = −0.10	R = −0.52	R = −0.45
	*p* = 0.754	*p* = 0.976	*p* = 0.438	*p* = 0.368	*p* = 0.413	*p* = 0.841	*p* = 0.20	*p* = 0.222

AD: atopic dermatitis; AR: allergic rhinitis; AC: allergic conjunctivitis.

## Data Availability

Data is contained within the article.
